# Donor lung weight a novel predictor for primary graft dysfunction

**DOI:** 10.1016/j.jhlto.2025.100271

**Published:** 2025-04-30

**Authors:** Andreas Martinsson, Anders Thoren, Sven-Erik Ricksten, Jonatan Oras, Moustafa Mohsen Abed, Petra Vestlund, Jesper M. Magnusson, Andreas Wallinder

**Affiliations:** a Department of Anaesthesiology and Intensive Care Medicine, Sahlgrenska Academy, University of Gothenburg, Gothenburg, Sweden; b Sahlgrenska University Hospital, Gothenburg, Sweden; c Department of Pulmonary Medicine, Sahlgrenska Academy, University of Gothenburg, Gothenburg, Sweden; d Unit for Organ Coordination, Transplant Institute, Sahlgrenska University Hospital, Gothenburg, Sweden; e Department of Molecular and Clinical Medicine, Institute of Medicine, Sahlgrenska Academy, University of Gothenburg, Gothenburg, Sweden; f Transplant Institute, Sahlgrenska University Hospital, Gothenburg, Sweden

**Keywords:** Donor lung weight, Primary graft dysfunction, ICU stay, Bilateral lung transplantation, Transplant complications

## Abstract

**Background:**

Primary graft dysfunction (PGD) remains a leading cause of early morbidity and mortality in lung transplantation. PGD is characterized by diffuse alveolar damage and the accumulation of extravascular lung water in the transplanted lung. Pre-existing injury and stress during the donation process are further aggravated by ischemia-reperfusion injury occurring during donation and transplantation. This study examines the relationship between adjusted donor lung weight, a surrogate for extravascular lung water, and outcomes following bilateral lung transplantation.

**Methods:**

We retrospectively analyzed 194 bilateral lung transplantations performed between January 2014 and May 2021. Donor lung weights were recorded after procurement, adjusted for body surface area, and categorized into quartiles. The primary outcomes assessed were the incidence of PGD (grades II and III) and duration of intensive care unit (ICU) stay. Secondary outcomes included mechanical ventilation duration, pulmonary function at discharge, and one-year mortality.

**Results:**

The incidence of PGD was significantly higher in the upper 4th quartile group ("high-weight," 22.9%) compared with the three lower quartile groups ("low-weight," 8.9%) (*p* = .020). Multivariate regression identified adjusted lung weight as an independent risk factor for PGD. The "low-weight" group showed higher rates of early extubation within 72 hours of lung transplantation (90.9% vs 83.0%, *p* = 0.048) and shorter ICU stays (median 3 vs 5 days, *p* = 0.026). No significant differences were found in ventilation duration, spirometry values, or 1-year survival.

**Conclusion:**

Adjusted donor lung weight is an independent predictor of PGD, suggesting that higher lung weights contribute to worse early outcomes post-transplant. Incorporating lung weight into donor assessment may improve recipient management and outcomes.

## Background

Lung transplantation offers improved survival and quality of life in end-stage pulmonary disease.^1^ While advancements in surgical techniques and post-transplant care have been remarkable, outcomes remain heavily reliant on the availability and quality of donor lungs.^2^, ^3^ Primary graft dysfunction (PGD) is a severe complication within 72 hours of lung transplantation and is the main determinant of early morbidity and mortality.^1^ Key factors in the etiology of PGD include the accumulation of extravascular lung water (EVLW) from adrenergic hyperactivity in donation after brain death and edema caused by ischemia-reperfusion injury.^1^, ^4^, ^5^ No donation after circulatory death (DCD) donors were included in our study cohort, as DCD became routine only after the recruitment period had concluded.

The overall incidence of PGD has risen^6^ to 40%, with severe forms (grade II and III) accounting for 15% to 30% of cases.^7^ PGD is the leading cause of early mortality in lung transplant recipients. It is also associated with adverse long-term outcomes, including chronic lung allograft dysfunction and more unfavorable survival.^6^, ^8^

Donor lung criteria have been established in a consensus document by the International Society for Heart and Lung Transplantation (ISHLT).^9^ However, donor lungs sustain injuries to varying and unpredictable degrees due to resuscitation maneuvers, mechanical ventilation, aspiration, or embolism.^10^ In clinical practice, ideal donor criteria are infrequently met. The etiology of PGD is multifactorial, involving a combination of donor, procedural, and recipient-related factors. Among donor characteristics, cigarette smoking has been identified as a specific risk factor for mild PGD, although not associated with increased mortality.^11^ On the recipient side, obesity, pre-transplant lung fibrosis, and pulmonary hypertension are significant risk factors for PGD.^12^

Improving the assessment of donor lungs and optimizing donor management protocols are critical strategies for reducing the incidence of PGD.^13^, ^14^, ^15^ In this study, we hypothesize that PGD grade II and III diagnosed within the first 72 hours postoperatively is associated with the adjusted lung weight at procurement.

## Materials and methods

### Study design, participant selection, and data acquisition

**Figure 1:**
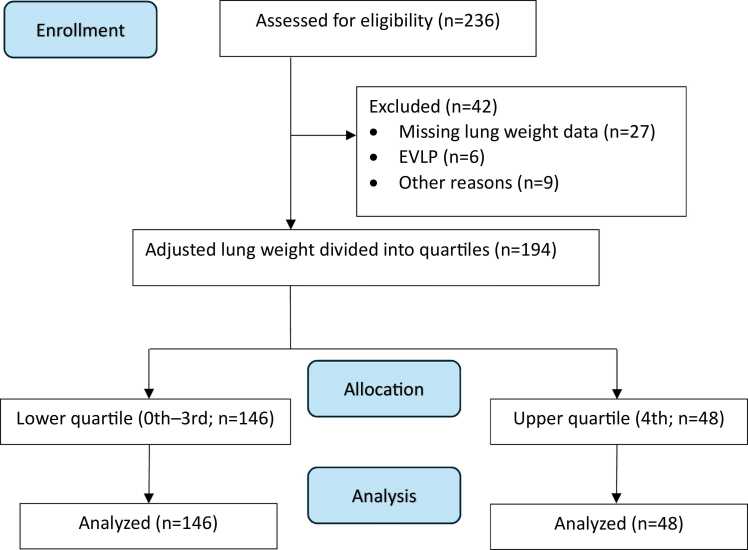
Study flowchart. All bilateral lung transplantations performed at Sahlgrenska University Hospital (Gothenburg, Sweden) between January 2014 and May 2021 were assessed for eligibility. Cases excluded for "other reasons" include bilateral lung lobe transplantations. EVLP, ex vivo lung perfusion.

The study was approved by the Swedish Ethical Board (Dnr: 2019-01473) and conducted in compliance with the ISHLT ethics statement. All patients provided informed consent for scientific investigations prior to transplantation. Donation after brain death donor lungs transplanted between January 2014 and May 2021 were identified using the Scandia transplant registry and assessed for eligibility (Figure 1). Exclusion criteria included lung lobe transplantation, the preoperative use of ex vivo lung perfusion (EVLP), and missing lung weight data. The guidelines from the ISHLT for grading PGD were applied.^4^ PaO_2_ and FiO_2_ values were obtained from laboratory records and patient charts. After weaning from mechanical ventilation, FiO_2_ was determined using a conversion table reflecting the amount of supplemental oxygen provided.^16^ Two experienced cardiothoracic radiologists reassessed all chest X-rays performed within the first 72 hours. In cases where a chest X-ray was unavailable, a plain scout image from a computed tomography scan was utilized.

### Transplant procedures

Adhering to a detailed protocol (Suppl), bilateral donor lung weights were measured and recorded following the standard clinical procurement and prior to the retrograde cold Perfadex flush. All measurements were consistently performed using the same digital table scale (GTIN: 04034127162040) on the back table at the donor site. Donor lungs were transported in cold storage on ice. The default surgical technique was sequential bilateral anterolateral thoracotomy without mechanical circulatory support. Perioperative veno-arterial (VA) ECMO support or cardiopulmonary bypass (CPB) were used selectively based on recipient hemodynamics (Table 1). A left-sided double-lumen endotracheal tube was used for single-lung ventilation, with lung-protective ventilation strategies employing tidal volumes of 6-8 mL/kg. The insertion of a Swan-Ganz catheter was routine for pulmonary artery pressure monitoring. During lung reperfusion, methylprednisolone (250 mg for each lung) and mannitol (100 mL for each lung) were administered. Anastomosis control was performed by perioperative bronchoscopy.

**Table 1:** Donor and Recipient Characteristics Divided by Weight Groups: Lower Quartile (Q1-Q3, “Low Weight”) and Upper Quartile (Q4, “High Weight”)

	Variable	Lower Quartile (Q1-Q3)	Upper Quartile (Q4)	*p* Value
		*n* = 146	*n* = 48	
Recipient data	Age, years (IQR)	58.5 (50.0-63.5)	59.3 (48.3-63.5)	0.971
	Year of transplantation	2017 (2016-2018)	2016 (2015-2018)	0.056
	Any MCS during transplantation, *n* (%)	41 (29)	13 (27)	1
	Cardiopulmonary bypass (CPB), *n* (%)	27 (18)	8 (17)	1
	CPB time, min (IQR)	197 (161-246)	242 (196-311)	0.163
	First lung, ischemia time, min (IQR)	257 (203-298)	246 (211-314)	0.644
	Second lung, ischemia time, min (IQR)	398 (315-472)	417 (340-506)	0.192
	Indication for transplantation: COPD, *n* (%)	32 (22)	9 (19)	0.69
	AAT, *n* (%)	12 (8)	4 (8)	1
	Pulmonary arterial hypertension, *n* (%)	8 (5)	3 (6)	1
	Cystic fibrosis, *n* (%)	14 (10)	2 (4)	0.366
	Fibrosis, *n* (%)	46 (32)	18 (38)	0.481
	Retransplantation, *n* (%)		3 (6)	0.411
	Sarcoidosis, *n* (%)	6 (4)	5 (10)	0.144
	Other, *n* (%)	23 (16)	4 (8)	0.237
Donor data	Age, years (IQR)	51 (35-64)	55 (42-66)	0.971
	Body Mass Index, kg/m²	24 (22-28)	24 (22-27)	0.947
	Smoking (any), *n* (%)	52 (36)	23 (48)	0.171
	Mechanical ventilation, hours (IQR)	48 (36-72)	48 (42-70)	0.93
	Aspiration (any), *n* (%)	18 (14)	7 (18)	0.606
	C-reactive protein (mg/L)	84 (38-159)	93 (45-145)	0.843
	White Cell Count (×10⁹/L)	12 (10-16)	14 (10-16)	0.426
	PaO_2_/FiO_2_ ratio at decision	60 (50-66)	58 (48-67)	0.569
	PaO_2_/FiO_2_ ratio pre-sternotomy	51 (41-60)	45 (37-57)	0.065
	PaO_2_/FiO_2_ ratio post-sternotomy	54 (43-61)	48 (39-60)	0.134
	Cause of death Intra Cerebral Hemorrhage, *n* (%)	75 (51)	32 (67)	0.068
	Anoxia, *n* (%)	31 (21)	10 (21)	1
	Trauma, *n* (%)	27 (18)	6 (13)	0.385
	Thrombosis, *n* (%)	6 (4)	0 (0)	0.339
	Other, *n* (%)	7 (5)	0 (0)	0.196
	Total Lung Capacity ratio pred.	1.03 (0.94-1.16)	1.00 (0.89-1.14)	0.323

AAT, Alpha-1 antitrypsin deficiency; Cause of death subheadings and their inclusions: Intra Cerebral Hemorrhage: ICH w/o spec., subarachnoid hemorrhage, subdural hemorrhage, cerebellar hemorrhage, and hemorrhage of the posterior cranial fossa; Anoxia: ischemic cerebral edema resulting from cardiac arrest; Trauma: head trauma, traumatic cerebral hemorrhage, and multi-trauma; Thrombosis: middle cerebral artery thrombosis, venous sinus thrombosis, and basilar artery thrombosis; Other causes: status epilepticus, drowning, hanging, meningitis, and paracetamol intoxication; COPD, chronic obstructive pulmonary disease; MCS, Mechanical Cardiac Support; Mechanical Ventilation: the duration of time the donor was on mechanical ventilation prior to aortic cross-clamping; Other indications for transplantation include: graft-versus-host disease, obliterative bronchiolitis, systemic scleroderma, lymphangioleiomyomatosis, and chronic pulmonary embolism; Smoking (any): indicates any history of smoking in the donor, regardless of duration, quantity, or whether the smoking was in the past; Total Lung Capacity ratio pred.: The ratio of donor to recipient predicted total lung capacity (TLC) for females derived using the formula “6.6 × height in meters − 5.97″ and the corresponding derivation for males “7.99 × height in meters − 7.08″.

Continuous variables reported as mean ± standard deviation or median with interquartile range (IQR), depending on data distribution. Categorical variables reported as *n* (%). Student's *t*-test for normally distributed variables and the Mann-Whitney *U* test for non-normally distributed variables. Categorical variables were compared using the chi-square test or Fisher's exact test, as appropriate.

Postoperative care was provided in the cardiothoracic intensive care unit (CICU), using a restrictive strategy for intravenous fluid administration along with continuous monitoring of perfusion and pulmonary hemodynamics (continuous cardiac output catheter, Edwards Lifesciences, Irvine, CA). Protective ventilation, including tidal volumes of 6-8 mL/kg of predicted body weight, peak pressure <30 cm H_2_O, and PEEP set to a minimum of 5 cm H_2_O (Flow-i; Maquet Critical Care, Solna, Sweden). Institutional protocols for weaning from mechanical ventilation were followed. According to the “Enhanced Recovery After Surgery” guidelines for cardiac surgery,^17^ early extubation was defined as occurring within 6 hours postoperatively. Immunosuppression therapy was initiated on the first postoperative day.

### Lung weight

To allow standardized comparisons across donors, lung weights were normalized for body size by dividing total bilateral donor lung weight by the donor's body surface area (BSA). BSA was calculated using the DuBois formula:^18^ BSA (m²) = 0.007184 × Weight (kg)^0.425 × Height (cm)^0.725. Data were categorized into lower (1st-3rd quartiles) and upper (4th) quartile groups and labeled as "low-weight" and "high-weight,” respectively. Limitations in existing scientific data on donor lung weights justify the decision to compare quartiles within the cohort.

### Primary and secondary outcomes

The primary outcomes were the incidence of PGD (grades II and III) at 24, 48, and 72 hours following reperfusion of the second lung and length of intensive care unit (ICU) stay. Secondary outcomes included the duration of postoperative mechanical ventilation, pulmonary function assessed by spirometry at hospital discharge, lung oxygenation over the first 72 hours, and mortality within the first year.

### Statistical analysis

Continuous variables were reported as mean ± standard deviation or median with IQR, depending on data distribution. The normality was assessed using the Shapiro-Wilk test. Student's t-test was used for normally distributed variables, and the Mann-Whitney *U* test for non-normally distributed variables. Categorical variables were compared using the chi-square test or Fisher's exact test, as appropriate. Trends across all quartiles were analyzed using the Mantel-Haenszel test for PGD severity and the Jonckheere-Terpstra test for ICU length of stay. Log-transformed values of adjusted donor lung weight were used in a linear regression model to identify variables associated with increased adjusted donor lung weight. Time to extubation and one-year mortality were evaluated using the Kaplan-Meier method, with the log-rank test employed to compare survival curves between groups. Multivariable logistic regression identified independent predictors of PGD. Variables with *p* < 0.1 in univariable analysis were included in the multivariable model. Results were presented as odds ratios (OR) with 95% confidence intervals (CI). For PaO_2_/FiO_2_ ratios, a repeated measures analysis of variance was employed to assess both the overall differences between the groups over time and to determine any significant interactions between the groups and time points. The PaO_2_/FiO_2_ ratio was dependent variable and time (0, 24, 48, and 72 hours) and group (lower quartiles and upper quartile) independent variables. Pairwise comparisons explored differences at individual time points. Statistical significance was set at *p* < 0.05. Statistical analyses were conducted using IBM® SPSS® Statistics software, Version 29.0 (IBM Corp., Armonk, NY).

## Results

### Patient demographics

Between January 2014 and May 2021, 236 bilateral lung transplants were performed at Sahlgrenska University Hospital (Gothenburg, Sweden), with 194 patients meeting all inclusion criteria (Figure 1). The most common indications for transplantation were pulmonary fibrosis (33.0%) and chronic obstructive pulmonary disease (21.1%; Table 1). Recipients ranged in age from 14 to 69 years, with a median age of 51 years. Sequential bilateral anterolateral thoracotomy was performed in 159 patients, with 19 requiring VA ECMO support. Thirty-five procedures were performed using CPB and median sternotomy. Donor lungs were implanted within a median time of 6.8 hours (IQR 5.3-8.1) following procurement.

The median adjusted donor lung weight for the entire cohort was 376.4 g/m² (IQR: 343.7-411.9 g/m²). In the lower three quartiles (*n* = 146), weights ranged from 224.8 to 479.7 g/m², while the upper quartile (*n* = 48) ranged from 480.7 to 1030.5 g/m². In CPB cases, bypass duration did not differ significantly between the groups. Similarly, the mean ischemia duration for the second donor lung (the last lung to be transplanted and reperfused) did not differ between groups. The donor mean PaO_2_/FiO_2_ ratio at the time of the initial decision to accept the lungs for transplantation was comparable across groups. However, the “high-weight” group exhibited a slightly lower PaO_^2^_/FiO_2_ ratio just before donor sternotomy (*p* = 0.065).

### Primary outcomes

A total of 24 patients (12.4%) were diagnosed with PGD grades II or III, including 13 (9%) in the “low-weight” group and 11 (23%) in the “high-weight” group (*p* = 0.020; Table 2). The median length of stay in the ICU was 3 days (IQR 2-5) for the “low-weight” group and 5 days (IQR 2-10) for the “high-weight” group (*p* = 0.026).

**Table 2:** Results Presented by Weight Groups: Lower Quartile (Q1-Q3, “Low Weight”) and Upper Quartile (Q4, “High Weight”)

Variable	Lower Quartile (Q1-Q3)	Upper Quartile (Q4)	*p* Value
	*n* = 146	*n* = 48	
Primary Graft Dysfunction II and III, *n* (%)	13 (9)	11 (23)	0.020
Cardiac Intensive Care Unit stay, *d* (IQR)	3 (2-5)	5 (2-10)	0.026
1st postop mechanical ventilation, hours (SD)	5 (3-14)	8 (4-33)	0.076
Total postop mechanical ventilation, hours (SD)	6 (3-18)	11 (4-132)	0.149
Postop extubation <6 hours, *n* (%)	82 (57)	20 (43)	0.095
Tracheostomy, *n* (%)	17 (12)	10 (21)	0.148
Sepsis, *n* (%)	8 (6)	3 (6)	1.000
Duration postop vasopressor or inotropy, *d* (IQR)	1 (1-3)	2 (1-5)	0.078
Vital Capacity (VC)_% pred._	65 (52-74)	65 (58-81)	0.367
Forced Expiratory Volume 1st second (FEV_1,0_)	2 (2-3)	2 (2-3)	0.490
FEV% (FEV_1,0_/FVC)	80 (73-88)	75 (70-87)	0.075

Duration postop vasopressor or inotropy: Duration of administration of any dosage of vasoconstrictor agents (eg, norepinephrine) and/or inotropic agents (eg, milrinone) during the postoperative period; Postop extubation <6 hours: Successful extubation achieved within 6 hours following surgery; Total postop mechanical ventilation: Cumulative duration of all postoperative invasive mechanical ventilatory support, including instances of reintubation and subsequent ventilation runs.

Continuous variables reported as mean ± standard deviation or median with IQR, depending on data distribution. Categorical variables reported as *n* (%). Student's *t*-test for normally distributed variables and the Mann-Whitney *U* test for non-normally distributed variables. Categorical variables were compared using the chi-square test or Fisher's exact test, as appropriate.

The trend across all four consecutive quartiles of adjusted donor lung weight was significant for PGD severity (*p* = 0.021), but not for ICU length of stay (*p* = 0.064, Table 3).

**Table 3:** **A.** Crosstabulation Across All Quartiles (Q1-Q4) Examining the Association Between PGD Severity (Grouped as 0-I vs II–III) and Adjusted Donor Lung Weight, Confirmed by a Statistically Significant Mantel-Haenszel Test for Trend (*p* = 0.021). **B.** ICU Length of Stay (Median Number of Days) Across All Quartiles (Q1-Q4)

**A.** Crosstabulation Between PGD Severity and Adjusted Donor Lung Weight					

					Total
Quartiles (Q)	Q1	Q2	Q3	Q4	
PGD 0-I	45	44	43	38	170
PGD II-III	3	5	5	11	24
Total	48	49	48	49	194

**B.** ICU Length of Stay					

Quartiles (Q)	Q1	Q2	Q3	Q4	
ICU stay (days)	3	2	3	4	

Quartiles are based on adjusted donor lung weight distribution, with the 1st quartile (Q1) representing the lowest 25th percentile and the 4th quartile (Q4) the highest.

ICU, intensive care unit; PGD, primary graft dysfunction.

The Jonckheere-Terpstra test for trend showed no significant association (*p = 0.064).*

### Secondary outcomes

**Figure 2:**
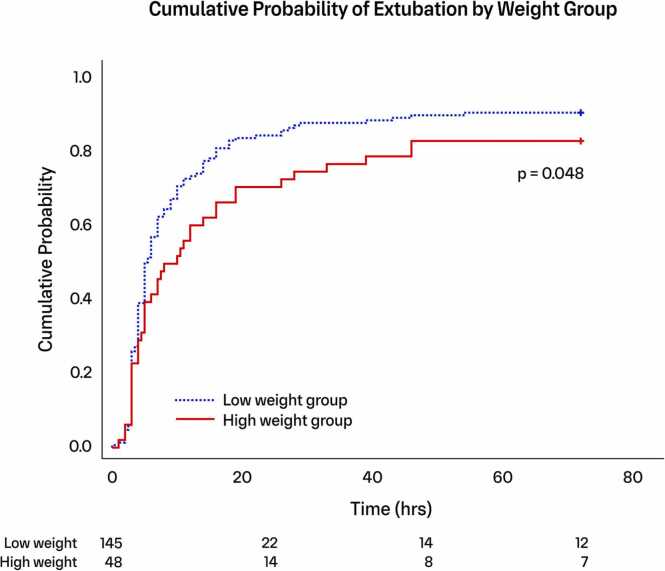
Kaplan-Meier plot illustrating the differences in the proportion of patients between groups regarding time to successful extubation within the first 72 postoperative hours. Log-rank test for group comparison. Numbers at risk shown at the bottom. The blue dotted line represents the “low weight” group, while the red solid line represents the “high weight” group.

A significantly higher proportion of patients in the "low-weight" group achieved successful extubation within the first 72 postoperative hours (*p* = 0.048; Figure 2). The mean duration of initial postoperative mechanical ventilation was 5 hours (IQR 3-14) in the “low-weight” group compared to 8 hours (IQR 4-33) in the “high-weight” group (*p* = 0.076; Table 2). When including reintubation and subsequent ventilation runs, the total duration was 6 hours (IQR 3-18) for the “low-weight” group and 11 hours (IQR 4-132) for the “high-weight” group (*p* = 0.149). Early extubation^17^ was achieved in 82 patients (57%) in the “low-weight” group compared to 20 patients (43%) in the “high-weight” group (*p* = 0.095). The duration of postoperative inotropic support was 1 day (IQR 1-3) in the "low-weight" group compared to 2 days (IQR 1-5) in the "high-weight" group (*p* = 0.078).

Measurement of lung function by spirometry was performed prior to hospital discharge, with a median time of 21 days (IQR 19-26) post-lung transplantation. While there was a difference in forced expiratory volume in one second (FEV_1,0_) as a percentage of forced vital capacity between the groups favoring the “low-weight” group [80% (73-88) vs 75% (70-87)], this difference was not statistically significant (*p* = 0.075). Furthermore, no significant differences were observed between the groups in terms of percent of predicted vital capacity or FEV_1,0_.

The multivariate regression analysis (Table 4) identified adjusted donor lung weight (*p* = 0.008) and shorter duration of mechanical ventilation prior to aortic cross-clamping in the donor (*p* = 0.013) as independent predictors associated with PGD. In a linear regression analysis assessing factors associated with increased adjusted donor lung weight (Table S1, Supplements), smoking was identified as the only variable with a statistically significant association.

**Table 4:** Multivariable Logistic Regression Analysis of Donor Patient Variables Conducted to Identify Independent Predictors of the Binary Outcome of Primary Graft Dysfunction Grade II and III (Present or Absent)

	Univariate			Multivariable		
Variable	OR	95% CI	*p* value	OR	95% CI	*p* value
Adjusted donor lung weight, per g/m²	1.004	1.00-1.01	0.004	1.004	1.00-1.01	0.008
Smoking (any)	1.698	0.72-4.01	0.227			
Duration Mechanical Ventilation, per hour	0.968	0.95-0.99	0.008	0.969	0.95-0.99	0.013
Age, per year	0.992	0.97-1.02	0.527			
Male sex	1.502	0.63-3.57	0.357			
Body Mass Index, per kg/m²	0.939	0.85-1.04	0.224			
Aspiration (any)	2.183	0.72-6.67	0.170			
PaO_2_/FiO_2_ ratio at decision	1.029	0.99-1.07	0.115			
Total Lung Capacity ratio pred.	1.336	0.22-8.04	0.751			

Duration Mechanical Ventilation: The total duration of mechanical ventilation until procurement; Total Lung Capacity ratio pred.: The ratio of donor to recipient predicted total lung capacity (TLC).

Variables with *p < 0.1 in the univariable analysis were included in the multivariable model.*

**Figure 3:**
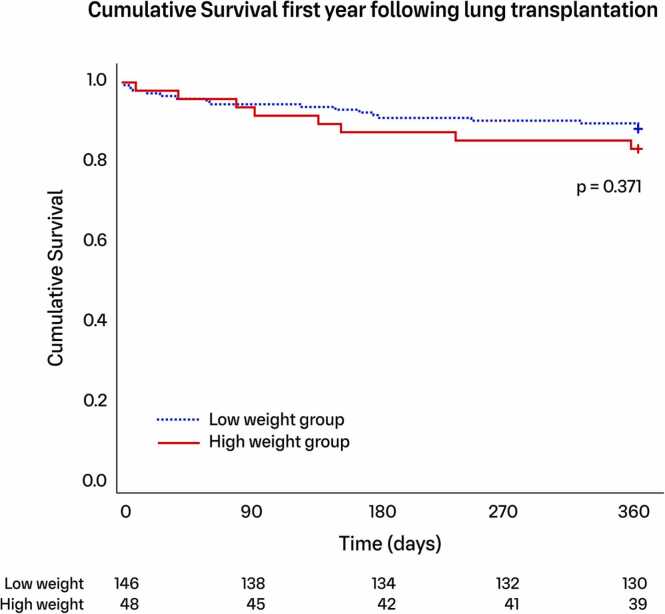
Kaplan-Meier plot illustrating the survival rate during the first year following lung transplantation. Log-rank test for group comparison. Numbers at risk shown at the bottom. The blue dotted line represents the “low weight” group, while the red solid line represents the “high weight” group.

**Figure 4:**
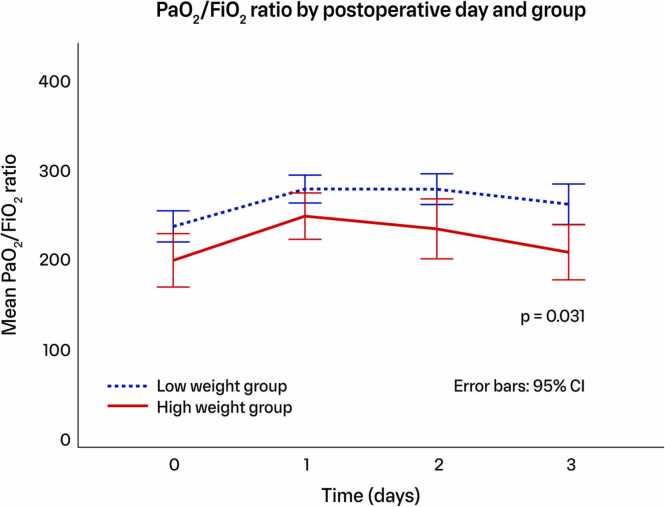
Graphical illustration of the mean PaO_2_/FiO_2_ ratio at 0, 24, 48, and 72 hours postoperatively, comparing the “high weight” group (red solid line) and “low weight” group (blue dotted line). Repeated measures analysis of variance (ANOVA) assessed overall differences in PaO_2_/FiO_2_ ratio between groups. Student's *t*-test comparisons at individual time points.

One year survival was 91.0% in the “low weight” group and 83.6% in the “high weight” group (*p* = 0.371, Figure 3). Postoperative mean PaO2/FiO2 ratios differed between the "high-weight" and "low-weight" groups in separation and trajectory of the curves over time (*p* = 0.031), with significant differences at 0, 48, and 72 hours (Figure 4).

## Discussion

This study identifies a significant association between adjusted donor lung weight and adverse outcomes, including the incidence of PGD grades II and III, as well as prolonged ICU stay. Recipients of heavier donor lungs experienced poorer clinical outcomes, reduced lung oxygenation, and prolonged extubation times within the first 72 hours post-transplant. These findings underscore the potential of donor lung weight as a surrogate marker for EVLW and a predictor of post-transplant complications. Awareness of donor lung weight can enable tailored recipient management strategies, potentially leading to improved patient outcomes.

In a study by Okamoto et al^19^ including 290 lung transplant recipients, 51 of whom received lungs treated with EVLP, the relationship between adjusted lung weight at procurement and the development of PGD grade III was investigated. A significant association was identified between the upper quartile of adjusted lung weight and PGD grade III in the combined cohort. However, this trend did not reach statistical significance in the non-EVLP subgroup (*n* = 239), likely due to lower lung weights and PGD risk.^13^ Our study excluded EVLP patients and is the first to demonstrate a significant association between adjusted donor lung weight and PGD incidence in non-EVLP cases, which continue to represent the majority of donor lungs. Excluding EVLP cases enhanced data consistency and allowed our findings to better reflect standard transplant procedures, minimizing the confounding effects of EVLP-related factors such as increased EVLW, variable organ quality, and differing treatment protocols. Ultimately, this approach strengthened the observed association between donor lung weight and PGD incidence.

Okamoto et al also reported longer ICU stays for recipients of heavier lungs, aligning with our findings. However, our study is the first to report a reduced rate of successful extubations within the first 72 hours among recipients of heavier donor lungs, consistent with significantly lower PaO_2_/FiO_2_ ratios. The prolonged ICU stays observed in the “high-weight” group in our study are likely linked to the increased incidence of PGD, which requires extended durations of mechanical ventilation. Prolonged ventilation, in turn, increases the risk of ventilator-associated pneumonia, delays patient mobilization, and negatively impacts cognitive recovery.^20^, ^21^

Interestingly, in the study by Okamoto et al, several patients improved from PGD grade III to grade II between 48 and 72 hours, likely due to effective postoperative treatment. Recognizing this dynamic nature of PGD grading, we used a composite measure of PGD grades II and III within the first 72 hours. This approach is further justified by the similar mortality risks associated with PGD grades II and III, as well as their shared link to an increased risk of Bronchiolitis Obliterans Syndrome,^8^ a significant long-term complication.

The association between increased donor lung weight and a higher incidence of PGD supports the hypothesis that heavier lungs have elevated levels of EVLW.^22^ This excess fluid adds to the perioperative ischemia-reperfusion injury edema and may also be a sign of more extended capillary damage.^23^ The autonomic storm associated with brain death induces neurogenic pulmonary edema and a surge in catecholamine release,^5^ which subsequently triggers pulmonary and systemic inflammatory responses. The inflammatory response to brain herniation varies due to genetic factors, and the balance of pro- and anti-inflammatory cytokines predicts recipient 30-day mortality.^24^, ^25^ Severe neurogenic pulmonary edema in donor lungs intensifies reperfusion injury and PGD risk in animal models.^26^ During donor management, the combined effects of inflammation and hemodynamic disturbances compromise the integrity of the capillary barrier, promoting accumulation of EVLW. Excessive volume resuscitation for vasoplegic vasomotor center dysfunction exacerbates the effect. Notably, the degree of lung weight gain observed during EVLP predicts lung suitability and post-transplant outcomes after 1 hour of EVLP.^27^ This finding likely reflects the persistence of severe capillary damage during donor care.

### Donor care

Our findings emphasize the need for tailored strategies to manage heavier donor lungs, including careful fluid management and appropriate use of diuretics to prevent fluid imbalance.^28^ Optimization of mechanical ventilation, including prone positioning for donors with hypoxemia, may improve ventilation-perfusion matching at allocation and reduce atelectasis, enhancing lung suitability for transplantation.^29^ In a group of postoperative cardiac surgery patients,^30^, ^31^ a brief period in the prone position combined with a mild recruitment maneuver effectively resolved atelectasis, leading to improved lung oxygenation and more homogeneous ventilation. This approach may also be viable for lung donors who cannot tolerate prolonged prone positioning.

### Postoperative care

Like donor management, negative fluid balance in postoperative care may minimize complications.^28^ Hoetzenecker et al^32^ reported a reduced PGD incidence with routine intraoperative ECMO, particularly citing potential benefits in heavier lungs. However, the absence of a comparison group limits causal interpretation, and a subsequent registry analysis found that ECMO increased PGD risk.^33^

The multivariate regression analysis identified adjusted donor lung weight as an independent determinant of increased PGD risk, consistent with the findings of Okamoto et al.^19^ Surprisingly, the analysis indicated that increased donor time on mechanical ventilation appeared to reduce the risk of PGD. This unexpected finding suggests that extended periods of mechanical ventilation may mitigate potential complications in the recipient. Possibly by removal of excess fluids from the donor lungs, enabling the resolution of pulmonary edema. Additionally, it may provide time for the donor to transition past the initial adrenergic storm typically associated with brain death.^26^ Moreover, extended mechanical ventilation may allow for optimization of ventilator settings, thereby improving lung function before procurement.

The PaO_2_/FiO_2_ ratio is a widely accepted variable for assessing donor lung quality, with values greater than 300 mmHg considered favorable.^1^, ^9^, ^10^ In this study, the donor PaO_2_/FiO_2_ ratio, measured at the time of decision as well as pre- and post-sternotomy, showed no significant differences between the "low-weight" and "high-weight" groups. This suggests that the PaO_2_/FiO_2_ ratio is not a reliable surrogate marker for lung-capillary damage in this context. McGiffin et al^34^ has previously demonstrated that the donor PaO_2_/FiO_2_ ratio alone is an insufficient predictor, leading to overly cautious decision-making and excessive rejection of donor lungs.

A significant difference was observed between groups in the postoperative PaO₂/FiO₂ ratio trajectory and specific time points. The “high-weight” group exhibited lower initial PaO₂/FiO₂ ratios, which remained lower and demonstrated less improvement over time. These findings align with the higher incidence of PGD in heavier lungs, as the PaO₂/FiO₂ ratio is a key determinant of PGD severity.

Although the reported combined incidence of PGD grades II and III is typically around 15%-30%,^7^ it was 12.4% in our cohort. This difference is unlikely due to patient selection, as most exclusions were related to missing lung weight data. Instead, it may be attributed to the relatively small cohort size, which increases susceptibility to random variation. Additionally, our strict criteria for diagnosing and grading PGD may have contributed to underdiagnosis, particularly in cases when clinicians, relying on clinical judgment, managed patients with low PaO_2_/FiO_2_ ratios without conducting immediate radiological tests. Furthermore, while our center’s surgical and perioperative care generally aligns with standard practices at larger transplant centers, certain aspects may differ. For instance, most procedures were performed without extracorporeal support, which likely reduced the inflammatory burden.^33^ Furthermore, the use of thoracic epidural anesthesia^35^ in patients without extracorporeal support may have reduced lung edema by enhancing endothelial integrity.^36^

The association between heavier donor lungs and increased PGD did not translate into significantly reduced 1-year survival despite the established link between PGD and early mortality. The absence of a significant difference in one-year mortality may stem from the lower-than-expected incidence of PGD grades II and III in our cohort.

### Limitations and future directions

The retrospective nature of the analysis limits causal inferences. In addition, we did not account for all potential confounding factors that could influence PGD risk and ICU stay duration. Moreover, the unexpectedly low overall incidence of PGD may limit the robustness of comparisons and affect the interpretation of outcomes.

In designing the study, we carefully considered whether to adjust lung weight for body height or BSA. Given the lack of conclusive evidence favoring one approach over the other, we ultimately selected BSA due to its broader representation of body size and its widespread use in medical research.

Comparing the upper lung weight quartile to the combined lower three quartiles was intended to maintain statistical power, simplify interpretation, and align with our aim to identify whether high lung weights are associated with increased PGD incidence. Focusing on a clearly defined high-risk group was considered more clinically relevant than examining incremental differences across all quartiles. This approach is further supported by the observed trend when analyzing quartiles as a continuum.

Future prospective studies should validate these findings and investigate interventions aimed at reducing donor lung weight. This includes optimizing donor management and refining the prediction of transplant suitability,^27^ potentially through the expanded use of EVLP. Incorporating prone positioning for donor lungs, with or without EVLP, and exploring the addition of hemofiltration are promising approaches to expand the donor pool.^29^, ^37^, ^38^, ^39^, ^40^ In situ normothermic regional perfusion for direct procurement and preservation may reduce the incidence of PGD.^41^ Furthermore, investigating the long-term impact of donor lung weight on outcomes such as chronic lung allograft dysfunction and overall survival is desirable.

## Conclusion

Our findings highlight the importance of considering adjusted donor lung weight in the selection and management of lung transplant recipients. The significant association between higher donor lung weight and increased PGD risk suggests that preoperative donor management strategies and selection of suitable lungs, as well as postoperative care tailored to recipients of heavier lungs, are crucial in optimizing outcome. Further studies with larger cohorts are needed to confirm and understand these findings.

## Funding

For this project, the authors received research grants from the Sahlgrenska University Hospital foundations (SU-961030, SU-971601), The Gothenburg Society of medicine (GLS-972306, GLS-999058), and the Swedish Heart-Lung Foundation (HjLfonden 20240788).

## Financial Disclosure Statement

On behalf of all authors of this manuscript, the authors confirm that none of the authors have any financial relationships or potential conflicts of interest to disclose in connection with this study.

## Declaration of Competing Interest

The authors declare that they have no known competing financial interests or personal relationships that could have appeared to influence the work reported in this paper.
