# Supplementary material for: Donor lung weight a novel predictor for primary graft dysfunction
Source: JHLT Open. 2025 Apr 30;9:100271. doi: 10.1016/j.jhlto.2025.100271 (PMC12166819; doi:10.1016/j.jhlto.2025.100271)
Supplement: Supplementary file 1 — Supplementary material [file mmc1.docx]

**Supplements**

**Protocol for Standardized Measurement and Recording of Bilateral Donor Lung Weight**

Lung procurement was performed by a small, consistent group of eight cardiothoracic surgeons from the same clinic, adhering to a well-established, standardized clinical procedure. Before explantation, the lungs were flushed with a low-potassium Dextran solution (Perfadex). The donor heart was explanted separately prior to lung explantation. No additional mediastinal tissue was included. The main bronchus was divided just proximal to the carina, and the lungs were placed in a tray on the back table without being separated. The cannula used for flushing was removed, and no retrograde flush was performed before weighing.

**Weighing Procedure**

1. **Scale Calibration and Preparation:** Before weighing, the digital scale was automatically calibrated. The empty tray used to hold the lungs during weighing was first placed on the scale and zeroed to ensure accurate measurement.
2. **Lung Weight Measurement:** The lungs were placed in the tray, and their total weight (in grams) was recorded and documented.
3. **Packing and Transport:** The lungs were packed in double plastic bags, securely sealed, and placed on ice. Insulation was added between the plastic layers to prevent frost damage during transport.
4. **Preparation for Transplantation:** Upon arrival in the transplant theater, the lungs were unpacked, subjected to a retrograde Perfadex flush, and prepared for anastomosis.

**Table S1**

| Factors associated with increased Adjusted Donor Lung Weight | | | | | | | | |  |  |
| --- | --- | --- | --- | --- | --- | --- | --- | --- | --- | --- |
|  |  |  | | | |  | |  | | |
|  |  | | B | Standard error | t | | p-value | | |  |
| CoD – Cerebrovascular Injury |  | | 0.029 | 0.017 | 1.744 | | 0.083 | | |  |
| CoD - Anoxia |  | | 0.002 | 0.020 | 0.113 | | 0.911 | | |  |
| CoD – Traumatic brain injury |  | | -0.030 | 0.022 | -1.374 | | 0.171 | | |  |
| CoD - Other |  | | -0.066 | 0.037 | -1.778 | | 0.077 | | |  |
| Body Mass Index |  | | 0.002 | 0.002 | 1.062 | | 0.290 | | |  |
| Smoking (any) |  | | 0.037 | 0.017 | 2.184 | | 0.030 | | |  |
| Diabetes |  | | -0.029 | 0.038 | -0.781 | | 0.436 | | |  |
| Mechanical ventilation (hours) |  | | 0.0002 | 0.0002 | -0.937 | | 0.350 | | |  |
| C-reactive protein (mg/L) |  | | 0.001 | 0.100 | 0.141 | | 0.888 | | |  |

Linear regression analysis examining possible factors associated with increased adjusted donor lung weight. Each factor was analyzed against the log-transformed value of adjusted donor lung weight.

The regression coefficient (B) represents the unstandardized effect size, indicating the change in log-transformed adjusted donor lung weight per unit change in the corresponding factor. The t-value reflects the ratio of B to its standard error, assessing the strength of the association. The p-value indicates statistical significance.

CoD: Cause Of Death
